# Ruxolitinib in IVIG-resistant kawasaki disease-associated macrophage activation syndrome complicated by a giant coronary artery aneurysm: a case report and narrative literature review

**DOI:** 10.3389/fped.2026.1977244

**Published:** 2026-10-30

**Authors:** Fan Zhang, Xuan Li, Ye Chen, Bo Wang, Ling Sun, Jie Huang

**Affiliations:** Department of Pediatric Cardiology, Institute of Pediatric Research, Children's Hospital of Soochow University, Suzhou, China

**Keywords:** giant coronary artery aneurysm, IVIG resistance, kawasaki disease, macrophage activation syndrome, ruxolitinib

## Abstract

**Background:**

Kawasaki disease (KD)-associated macrophage activation syndrome (KD-MAS) is a life-threatening complication with limited treatment options. Ruxolitinib, a JAK1/2 inhibitor, has shown efficacy in refractory systemic juvenile idiopathic arthritis-associated MAS (sJIA-MAS) and hemophagocytic lymphohistiocytosis (HLH). Its use in KD-MAS has been described in one previous case treated with ruxolitinib plus corticosteroids; use in a patient who also developed a giant coronary artery aneurysm has not been reported. A 3-month-old male infant with IVIG-resistant KD developed persistent fever despite two courses of IVIG, the first given before referral, and methylprednisolone pulse therapy. KD-MAS was diagnosed on the basis of bicytopenia, hyperferritinemia, elevated soluble CD25, hypofibrinogenemia, and hemophagocytes in the bone marrow. Ruxolitinib was initiated early. Body temperature normalized within 72 h, with rapid improvement in inflammatory markers. Coronary dilation was documented on illness day 22. By illness day 24, the left anterior descending artery (LAD) met giant-aneurysm criteria (*Z*-score: 10.93; later peak 11.9), whereas the proximal right coronary artery remained a medium aneurysm (peak *Z*-score: 5.73). After a third course of IVIG and long-term antithrombotic therapy with aspirin plus low-molecular-weight heparin, the aneurysms regressed over 16 months of follow-up. Clinical and laboratory improvement temporally followed the addition of ruxolitinib, although the contribution of ruxolitinib cannot be separated from concomitant IVIG and glucocorticoid therapy.

**Discussion:**

A narrative literature review identified ten relevant studies. Reported KD-MAS treatment is predominantly IVIG plus glucocorticoids, with cyclosporine A and biologics reserved for refractory cases. This case is the second published report of ruxolitinib in KD-MAS, and the first to document a giant coronary artery aneurysm with 16-month coronary follow-up.

**Conclusion:**

KD-MAS should be suspected in IVIG-resistant KD with persistent fever and cytopenias. Addition of ruxolitinib was temporally associated with rapid clinical and inflammatory-marker improvement; however, its independent therapeutic contribution cannot be determined because of concurrent IVIG and glucocorticoid therapy. Systemic inflammatory improvement did not prevent coronary progression, so intensive coronary surveillance must continue.

## Introduction

Kawasaki disease (KD) is an acute medium-vessel vasculitis that mainly affects children under 5 years of age. Without prompt treatment, up to a quarter of patients develop coronary artery abnormalities, and KD is now the leading cause of acquired heart disease in children (1, 2). Macrophage activation syndrome (MAS) is a life-threatening hyperinflammatory state driven by aberrant macrophage activation and a cytokine storm; it lies within the spectrum of secondary hemophagocytic lymphohistiocytosis (HLH) and is classically associated with systemic juvenile idiopathic arthritis (sJIA), but may also complicate KD (KD–MAS) (3).

KD–MAS has an insidious onset but a rapid course, and no internationally agreed diagnostic criteria or large epidemiological estimates are available. Recent reviews place its incidence at roughly 1%–2% among children with KD, rising appreciably in IVIG-resistant disease (4, 5). Once MAS supervenes, coronary injury, multiorgan failure and death are far more common. It is distinguished from primary/familial HLH, infection- or sepsis-associated hyperinflammation, and severe KD with consumptive coagulopathy by its onset during the acute KD phase, the absence of an identifiable infectious trigger, and an sJIA–MAS-like phenotype superimposed on classic coronary vasculitis.

Mechanistically, KD–MAS reflects an excess of proinflammatory M1 macrophages with impaired M2-type resolution, together with dysfunctional NK cells and cytotoxic T lymphocytes; the resulting surge of IFN-*γ*, TNF-α, IL-1β, IL-6 and IL-18 sustains a self-amplifying inflammatory loop. Treatment is still non-standard and largely extrapolated from sJIA–MAS—typically IVIG and glucocorticoids, with cyclosporine A or biologics added in refractory cases—and some patients remain systemically inflamed despite these measures (6).

Ruxolitinib, a selective JAK1/2 inhibitor, blocks JAK–STAT signaling and thereby dampens downstream signaling of several proinflammatory cytokines, including IFN-*γ* and IL-6; it has been used as salvage therapy in refractory sJIA–MAS and HLH (7). Its use in KD-MAS remains limited to a single prior report of ruxolitinib combined with corticosteroids. We describe an infant with IVIG-resistant KD-MAS who developed a giant LAD aneurysm and medium-sized aneurysms of the left main and right coronary arteries, received early ruxolitinib, and was followed for 16 months. We also narratively review the pathogenesis, diagnosis, and treatment of KD-MAS.

## Narrative literature review

This was a narrative review, not a systematic review. PubMed, Wanfang, and other Chinese and English databases were searched, without language or date restrictions, using the terms “ruxolitinib,” “Kawasaki disease,” “macrophage activation syndrome,” “giant coronary artery aneurysm,” and “IVIG resistance.” The search was used to identify representative publications on the clinical features, treatment, and outcomes of KD-MAS; consensus statements or case reports on refractory MAS; and experience with ruxolitinib or other JAK inhibitors in MAS/HLH. Ten studies were selected for discussion and are summarized in Table 1. No claim of exhaustive retrieval is made.

**Table 1:** Studies discussed in the narrative review of Kawasaki disease-associated macrophage activation syndrome (KD-MAS).

Authors	Year	Study Type	Cases (n)	Key Findings/Therapeutic Regimens	Prognosis
Inguscio et al. (4)	2025	Systematic literature review	176	Fever 100%, splenomegaly 49.4%, hyperferritinemia 98.2%, cardiac involvement 37%; glucocorticoids 82.8%, cyclosporine 28.7%, biologics 13.8%; HLH-2004 protocol 20%	Improved
Pilania et al. (5)	2021	Retrospective case series	12	KD-MAS accounted for 1.3% of all KD cases; all were IVIG-resistant	All improved
Baldo et al. (6)	2025	Systematic review	69	KD-MAS accounted for ∼6.0% of all MAS cases; treatment mainly glucocorticoids plus IVIG; high data heterogeneity	—
Wang X et al. (8)	2026	Case report	1	Ruxolitinib combined with corticosteroids in KD-MAS; rapid clinical response	Improved
Shakoory et al. (9)	2023	Guideline consensus (EULAR/ACR)	—	Ruxolitinib and other agents recommended for refractory cases (under clinical trials)	—
García-Pavón et al. (10)	2017	Systematic review (include 2 case reports)	69	Males 68%, median age 5.6 years; glucocorticoids 87%, cyclosporine 49%	High mortality
Wen et al. (11)	2023	Retrospective case-control analysis	27	Hepatosplenomegaly, IVIG resistance, and coronary artery lesions significantly more frequent than in KD alone	Prolonged hospital stay
Jeong & Lee (12)	2025	Literature review	23	Incidence of KD-MAS 0.8%–1.1%; glucocorticoids 100%, cyclosporine 74%, etoposide 70%, infliximab 4%	∼2/3 improved
Liu et al. (13)	2026	Case report with literature review	2 (sJIA-MAS)	Ruxolitinib 10 mg twice daily plus plasma exchange; fever resolved in 2–4 days; glucocorticoids tapered off in 3–6 months	Improved
Li et al. (14)	2025	Retrospective study	20 (adult RD-MAS)	Ruxolitinib improves prognosis of refractory MAS	Partial response

KD-MAS, Kawasaki disease-associated macrophage activation syndrome; IVIG, intravenous immunoglobulin; sJIA-MAS, systemic juvenile idiopathic arthritis-associated macrophage activation syndrome; RD-MAS, rheumatic disease-associated macrophage activation syndrome; HLH, hemophagocytic lymphohistiocytosis; —, not applicable or not reported.

### Case presentation

This study was approved by the Medical Ethics Committee of the Children's Hospital of Soochow University (approval no.2026CS393). Informed consent was obtained from the patient's guardians.

### Clinical data

A 3-month-3-day-old term boy (G1P1, spontaneous vaginal delivery) was admitted on illness day 7 with fever, rash, and extremity edema; fever had already lasted 7 days. The first IVIG course, a single total dose of 2 g/kg, had been given at the referring hospital on illness days 5–6, before transfer. On arrival, temperature was 37.8° C, heart rate 137 bpm, respiratory rate 20 breaths/min, and SpO_2_ 98% on room air. He was lethargic, with a scattered erythematous rash, cheilitis, and hand–foot edema.

By illness day 9 he remained febrile, with persistent bicytopenia, rising ferritin and sCD25, and falling fibrinogen. A second IVIG course (2 g/kg as a single total dose on illness days 9–10), methylprednisolone pulse therapy (10 mg/kg/day on days 9–11) followed by a 2-week oral prednisone taper, aspirin (30–50 mg/kg/day), and a washed red-cell transfusion were administered. On day 11, fever persisted, fibrinogen fell to 0.997 g/L, and marrow aspiration showed hemophagocytes. KD-MAS was diagnosed according to the 2020 Chinese expert consensus on KD-MAS. The presentation was also consistent with the HLH-2004 diagnostic framework and was considered against the 2022 EULAR/ACR points to consider for suspected HLH/MAS. Primary familial HLH and infection-associated hyperinflammation were considered but not supported. Ruxolitinib was then started at 2.5 mg every 12 h (approximately 0.77 mg/kg/day in this 6.5 kg infant), a weight-adjusted pediatric salvage dose based on published infantile HLH/MAS experience and institutional protocol, without further titration. Fever resolved and CRP fell to 7 mg/L within 72 h.

On day 22, echocardiography showed dilation of both proximal coronary arteries, and nadroparin was added to aspirin. By day 24, the LAD met giant-aneurysm criteria (*Z*-score: 10.93; peak 11.9 on day 31). The proximal RCA did not: its peak *Z*-score was 5.73 and its absolute diameter was 3.1 mm, meeting criteria for a medium aneurysm but not for a giant aneurysm (*Z*-score ≥ 10 or absolute diameter ≥ 8 mm). The left main coronary artery likewise remained medium (peak *Z*-score: 5.39). A third IVIG course (2 g/kg as a single total dose) was given on illness day 24, and aspirin plus nadroparin calcium was continued. He was afebrile and stable by day 37 and was discharged. Ruxolitinib was prescribed from illness day 11 through day 37 (about 26 days), including a 5-day interruption for respiratory syncytial virus infection, as described below. Long-term aspirin and nadroparin were continued (Table 2; Figures 1, 2). Defervescence and normalization of inflammatory markers did not stop coronary enlargement: between days 22 and 24 the LAD enlarged into the giant range, while the proximal RCA remained below that threshold. Serial echocardiography therefore has to continue after systemic inflammation has settled (Table 2).

**Table 2:** Summary of concurrent medications during hospitalization.

Medication	Dose	Started (illness day)	Stopped (illness day)	Indication
IVIG, 1st course (referring hospital)	2 g/kg,single total dose	5	6	Initial KD
IVIG (2nd round)	2 g/kg,single total dose	9	10	IVIG-resistant KD
IVIG (3rd round)	2 g/kg,single total dose	24	24	Giant coronary aneurysms
Methylprednisolone	10 mg/kg/day	9	11	Refractory fever/MAS
Prednisone	Oral taper	After day 11	2-week taper	Steroid taper
Ruxolitinib	2.5 mg q12h (0.77 mg/kg/day)	11	37,with a 5-day hold	KD-MAS salvage
Aspirin	30–50 then 3–5 mg/kg/day	9	ongoing	Antiplatelet
Nadroparin	100 IU/kg q12h	22	∼3 months after discharge	Anticoagulation
Washed RBC transfusion	1 unit	9	9	Severe anemia

**Figure 1:**
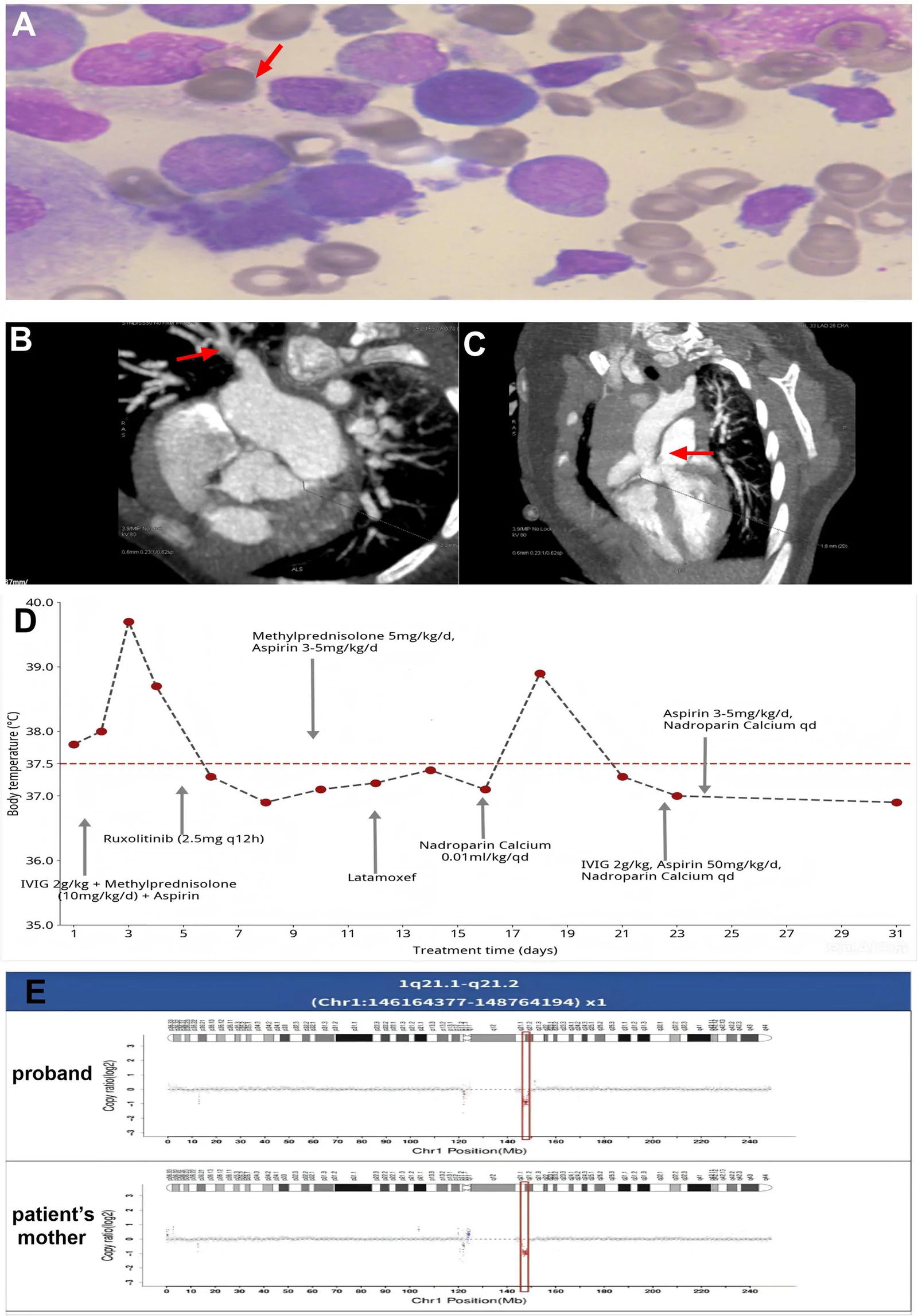
Clinical characteristicsn, bone marrow findings, cardiac imaging, and in-hospital temperature-medication timeline of the patient. **(A)** Bone marrow smear showing hemophagocytes. **(B,C)** Cardiac computed tomography angiography showing aneurysmal involvement of the left and right coronary arteries. Only the LAD met giant-aneurysm criteria; the proximal RCA was medium-sized. The affected segments are labelled (LMCA/LAD and RCA).**(D)** Genetic test results (1: proband; 2: patient's mother). **(E)** Timeline of body temperature and major medications, including pre-referral IVIG on illness days 5–6. The red dashed line indicates the upper limit of normal body temperature.

**Figure 2:**
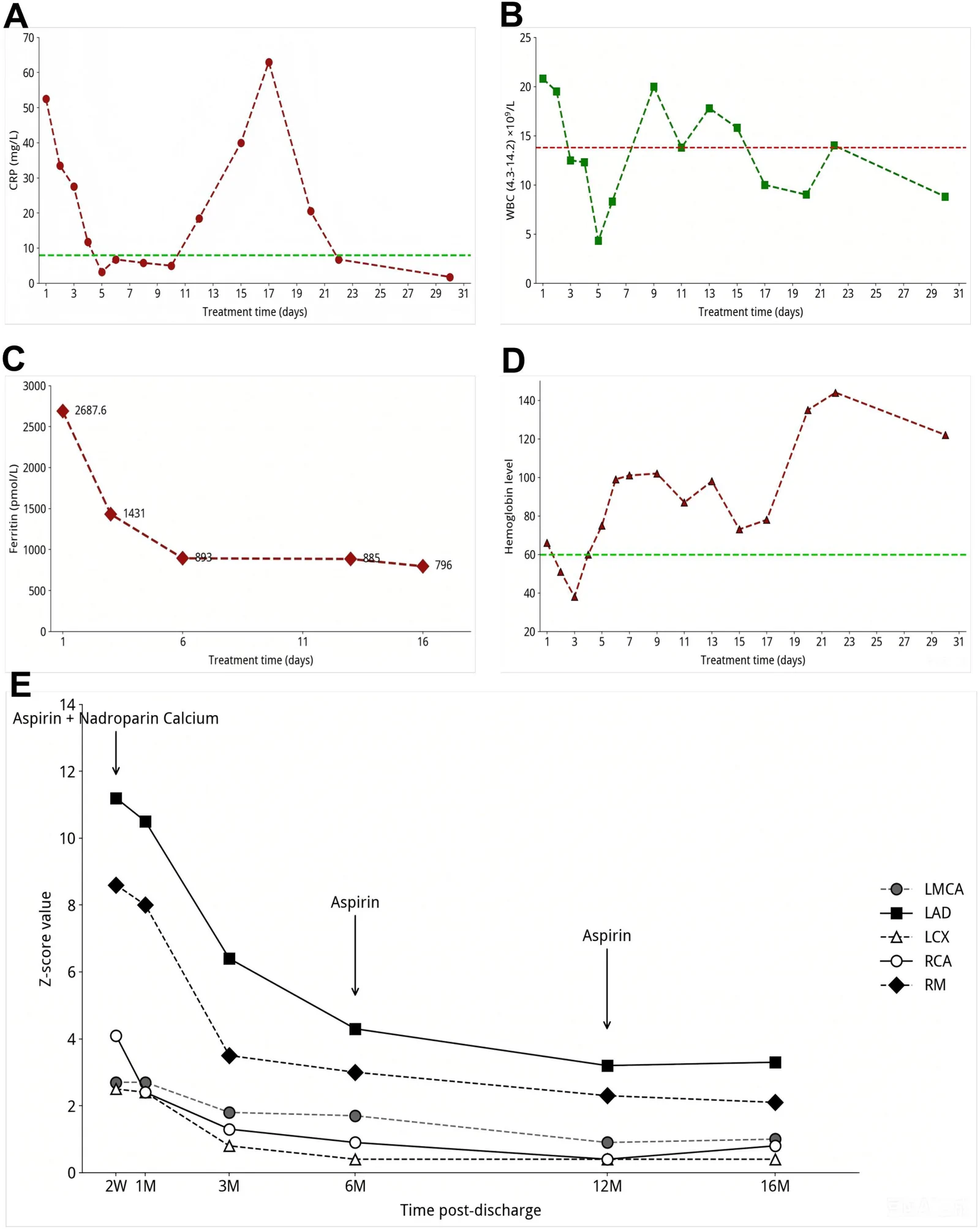
Trends of laboratory parameters and coronary artery *Z*-scores during hospitalization and follow-up. **(A)** Serial C-reactive protein curve. **(B)** Changes in white blood cell count. **(C)** Dynamic changes in serum ferritin. **(D)** Dynamic changes in hemoglobin. **(E)** Follow-up trends of *Z*-scores for each coronary artery branch after discharge. Serial platelet count, fibrinogen, AST/ALT, triglycerides and D-dimer have been added to reflect the evolution and resolution of MAS.

### Follow-up

The infant was followed for 16 months after discharge (to 25 months of age). Subcutaneous nadroparin calcium (100 IU/kg q12h) was given for 3 months, after which the aneurysms began to regress and showed marked improvement by 6 months. Low-molecular-weight heparin was then discontinued given substantial aneurysm reduction and stable *Z*-scores, and aspirin (3–5 mg/kg/day) was continued. Serial echocardiography showed progressive reduction of the coronary aneurysms, including the giant LAD aneurysm, with falling *Z*-scores (Tables 3, 4). The LAD *Z*-score was still above 3 (3.36) at 16 months, indicating regression from its previous giant dimension but residual dilation; ventricular systolic function and ECG remained normal. The LAD *Z*-score remained above 3 (3.36) at the 16-month visit, suggesting regression of the coronary arteries from their previous giant dimensions.

**Table 3:** Coronary artery diameter and *Z*-score at different treatment time points.

Illness day	LMCA (mm/*Z*-score)	LAD (mm/*Z*-score)	LCX (mm/*Z*-score)	RCA (mm/*Z*-score)	Mid-segment (mm/*Z*-score)
Illness day 9	2.2/2.29	1.9/2.59	1.5/1.48	2/2.33	1.7/2.07
Illness day 22	3/4.8	4.1/9.9	1.8/2.46	2.6/4.18	2.5/4.6
Illness day 24	3.2/5.39	4.4/10.93	1.8/2.46	3.1/5.7	3.5/7.77
Illness day 27	3/4.77	4.4/10.93	1.7/2.13	3.1/5.72	3.6/8.09
Illness day 31	2.9/4.46	4.7/11.9	1.9/2.78	3.1/5.72	3.6/8.09
Illness day 37	2.4/2.91	4.7/11.9	1.9/2.78	2.8/4.8	3.9/9

LMCA, left main coronary artery; LAD, left anterior descending artery; LCX, left circumflex artery; RCA, right coronary artery. *Z*-score > 2 indicates coronary artery dilation; *Z*-score in red. Dimensions were measured at end-diastole on the parasternal short-axis view by experienced pediatric echocardiographers; *Z*-scores are body-surface-area-adjusted and calculated with the Boston Children's Hospital (McCrindle/Lipshultz) reference equations; values are echocardiographic, with CT angiography used only for morphological confirmation. Illness days are counted from the first day of fever.

**Table 4:** Coronary artery diameter and *Z*-score during follow-up.

Follow-up time	LMCA (mm/*Z*-score)	LAD (mm/*Z*-score)	LCX (mm/*Z*-score)	RCA (mm/*Z*-score)	Mid-segment (mm/*Z*-score)
2 weeks post-discharge	2.3/2.75	2.3/11.14	1.8/2.6	2.50/4.04	3.70/8.63
1 month post-discharge	2.3/2.75	2.3/10.47	1.8/2.6	2.00/2.48	3.50/7.99
3 months post-discharge	2.3/1.87	2.3/6.39	1.5/0.88	1.90/1.35	2.40/3.50
6 months post-discharge	2.3/1.7	2.3/4.31	1.4/0.44	1.80/0.91	2.30/3.04
12 months post-discharge	2.2/0.96	2.2/3.23	1.6/0.66	1.80/0.50	2.20/2.29
16 months post-discharge	2.3/1.07	2.3/3.36	1.6/0.53	2.00/0.90	2.20/2.14

LMCA, left main coronary artery; LAD, left anterior descending artery; LCX, left circumflex artery; RCA, right coronary artery. *Z*-score > 2 indicates coronary artery dilation; *Z*-score in red. *Z*-scores were calculated with the same BSA-adjusted reference system and by the same observer(s) as the in-hospital measurements. The persistent LAD *Z*-score > 3 at 16 months indicates residual abnormality despite substantial regression.

### Genetic testing

Chromosomal microarray analysis (CMA) detected a 2.6 Mb heterozygous deletion at chr1:146164377-148764194 (1q21.1-q21.2) (see Figure 1D). Family verification confirmed that the variant was inherited from the mother. No known KD or MAS pathogenic genes were identified within the deleted interval; neither the patient nor the mother exhibited typical features of 1q21.1 deletion syndrome. No published reports have directly linked this copy number variation to KD or KD-MAS, and it is considered an incidental genetic finding.

## Discussion

We report an infant of 3 months with IVIG-resistant KD–MAS who received ruxolitinib, and document the coronary course to 16 months. Ruxolitinib has been reported in KD-MAS only once before (8), and no previous account has combined that exposure with a giant coronary artery aneurysm and long-term coronary follow-up.

Fever and inflammatory markers settled rapidly once ruxolitinib was added—within 72 h fever resolved and CRP fell to 7 mg/L—on top of two IVIG courses and methylprednisolone pulses. That systemic improvement did not protect the coronary arteries. The LAD continued to enlarge to a giant aneurysm between illness days 22 and 24, whereas the proximal RCA remained a medium aneurysm, before the lesions regressed on long-term antithrombotic therapy.

KD–MAS is recognized mainly through evolving laboratory abnormalities rather than any single test. The larger reviews broadly agree on the typical picture—persistent fever, marked hyperferritinemia, splenomegaly and a substantial rate of coronary involvement, almost always in children already resistant to IVIG (4, 10–12)—and on the clue most relevant here: cytopenia that worsens rather than improves. Our patient was younger than the usual reported age and, unlike the reactive thrombocytosis of acute KD, he was already anemic and thrombocytopenic, with low fibrinogen, before any steroid was given. When these were followed by rising ferritin and sCD25, falling fibrinogen and marrow hemophagocytes, the case met the 2020 consensus criteria for KD–MAS. An IVIG-resistant, febrile child whose platelets fall rather than rise should therefore be investigated for MAS without delay.

Treatment remains extrapolated from sJIA–MAS and is not standardized. Across the literature, IVIG combined with glucocorticoids forms the backbone, with cyclosporine A and biologics reserved for refractory disease; IL-1 and IFN-*γ* blockade have shown benefit in sJIA–MAS, but evidence in KD–MAS remains scant (6). Before this report, ruxolitinib had been used in KD–MAS in a single patient, together with corticosteroids (8); elsewhere it produced rapid fever control in refractory sJIA–MAS and some benefit in adult rheumatic-disease MAS (13, 14). The present case therefore adds the second published KD-MAS exposure to ruxolitinib and, unlike the first, follows a giant LAD aneurysm for 16 months.

The 72 h defervescence cannot be attributed to ruxolitinib alone, since IVIG and glucocorticoids were continued throughout; it falls within the range reported for ruxolitinib-treated sJIA–MAS but is equally compatible with the cumulative effect of two IVIG courses and pulse steroids. The admission cytokine panel showed raised IFN-*γ*, IL-6 and IL-10 with IL-18 within range; because IFN-*γ* and IL-6 signaling both proceed through JAK1/JAK2, this offers at least a plausible substrate for upstream JAK blockade. That said, this is a single sample taken at presentation: it hints at an IFN-*γ*/IL-6-skewed phenotype but cannot define one, and provides no basis on which to prefer ruxolitinib over anakinra, infliximab or emapalumab. JAK inhibitors have also been explored in multisystem inflammatory syndrome in children (MIS-C), another post-infectious cytokine storm in which dysregulated JAK-STAT signaling drives hyperinflammation (15); this convergent biology further supports the rationale for JAK-STAT blockade in KD-MAS, although direct comparative evidence is lacking (Table 5).

**Table 5:** Serum cytokine profile at admission.

Cytokine	Reference range (pg/mL)	Result (pg/mL)
GM-CSF	0–39	158.7 ↑
GRO-alpha	0–93	19.6
IFN-alpha	0–0.2	3.7 ↑
IFN-gamma	0–95	138.6 ↑
IL-2	0–74	211.8 ↑
IL-4	0–45	45.6 ↑
IL-5	0–17	59.5 ↑
IL-6	0–51	134.9 ↑
IL-7	0–8	9 ↑
IL-8	0–47	13.9
IL-9	0–6	147.4 ↑
IL-10	0–3	97.2 ↑
IL-15	0–22	36 ↑
IL-17A	0–53	26.4
IL-18	0–957	149.4
IL-21	0–143	192.7 ↑
IL-23	0–8	219 ↑
IL-27	0–220	186.9
IL-31	0–9	126 ↑
MIP-1 beta	0–147	149.2 ↑
TNF-beta	0–14	39.4 ↑

Values above the reference range are marked with ↑ (red). IFN, interferon; IL, interleukin; GM-CSF, granulocyte-macrophage colony-stimulating factor; GRO, growth-regulated oncogene; IP, interferon-inducible protein; MCP, monocyte chemoattractant protein; MIP, macrophage inflammatory protein; RANTES, regulated on activation normal T cell expressed and secreted; SDF, stromal cell-derived factor; TNF, tumor necrosis factor. Cytokines were measured by flow cytometric bead array (Luminex xMAP); reference ranges are laboratory-adapted pediatric intervals for age.

The clinically most important point is that the systemic and coronary responses moved on different timetables. Fever and CRP normalized within days, yet the LAD continued to dilate to a giant aneurysm, with the *Z*-score peaking above 11, on illness days 22 to 24. The proximal RCA never entered the giant range. Rapid defervescence therefore cannot be taken as evidence that coronary risk has passed; the lag between the two means echocardiographic surveillance has to continue regardless of how the patient looks. With aspirin plus nadroparin, the aneurysms then regressed, though unevenly: by 16 months the LMCA and RCA *Z*-scores had returned close to normal, while the LAD remained mildly enlarged (Z 3.36; Figure 2E). Cases of refractory KD with rapidly enlarging giant aneurysms describe long-term antithrombotic therapy, sometimes combined with beta-blockade or ACE inhibition, as a way to stabilize aneurysms during convalescence (16, 17). Our case fits and adds a caution: the most severely affected segment may not fully resolve even after neighboring segments do, so a single normal branch is not grounds to stop surveillance.

The drug was otherwise well tolerated. A lower respiratory RSV infection appeared within a week of starting it, with fever, cough and a transient CRP rise; ruxolitinib was held for five days and the episode resolved with supportive treatment, without neutropenia or lymphopenia. On timing, this episode is more likely infectious than a flare of MAS (Figure 1D). It nonetheless illustrates the infection risk of JAK inhibition and the need to monitor blood counts and liver enzymes during treatment.

The maternally inherited 2.6 Mb deletion at 1q21.1–q21.2 was incidental: neither the child nor his mother showed a 1q21.1 phenotype, and the interval contains no known KD- or MAS-related gene. We do not assign it a causal role, although a modifying effect of an immune-related gene in the region cannot be excluded.

### Study limitations

This is a single, uncontrolled case and carries several limitations. IVIG, glucocorticoids and ruxolitinib were administered concurrently, so the independent contribution of ruxolitinib cannot be separated; serial cytokine measurements and pharmacokinetic data are also lacking, and the infant dose used here remains empirical. The accompanying literature summary is a narrative review of heterogeneous sources. It was not conducted or reported as a systematic review.

## Conclusion

KD–MAS is a life-threatening complication of KD, and current treatment remains based mainly on IVIG combined with glucocorticoids. In this infant, adding ruxolitinib was followed by rapid defervescence and improvement in inflammatory markers; although its independent effect cannot be distinguished from that of concurrent IVIG and glucocorticoids, we consider it likely that ruxolitinib made a beneficial contribution to the treatment of KD–MAS in this case.

## Data Availability

The original contributions presented in the study are included in the article/Supplementary Material, further inquiries can be directed to the corresponding author/s.
